# A Combination of Two Variants p. (Val510 =) and p. (Pro2145Thrfs * 5), Responsible for von Willebrand Disease Type 3 in a Caribbean Patient

**DOI:** 10.1055/s-0040-1718703

**Published:** 2020-10-27

**Authors:** Marie Daniela Dubois, Serge Pierre-Louis, Johalène Rabout, Cécile V. Denis, Olivier Christophe, Sophie Susen, Jenny Goudemand, Pierre Boisseau, Rémi Neviere, Olivier Pierre-Louis

**Affiliations:** 1 EA 7525 VPMC, Université des Antilles, Schoelcher, Martinique, France; 2 Ressources and Competence Center for Constitutional Hemorrhagic Diseases (CRC-MHC), CHU Martinique, Martinique, France; 3 HITh, UMR_S1176, INSERM, Université Paris-Saclay, Le Kremlin-Bicêtre cedex, France; 4 Department of Hematology and Transfusion, CHU Lille, Lille, France; 5 Department of Medical Genetics, Hôtel-Dieu Hospital, CHU Nantes, Nantes, France; 6 Fort-de-France, CHU Martinique, Martinique, France

von Willebrand disease (VWD) represents one of the most common inherited hemorrhagic disorders in France with 1,980 patients identified in the FranceCoag network in December 2016. 1 The disease results from genetic defects generally localized in the von Willebrand factor ( *VWF* ) gene, defects that can either modify the function of the protein or affect its clearance and/or synthesis. In the French Caribbean island of Martinique, VWD prevalence in symptomatic subjects amounts to approximately 0.02% of the population. This work describes a new variant p.(Val510 = ), located in the D2 domain of VWF, in Martinican's families. This variant p.(Val510 = ) associated with the variant p.(Pro2145Thrfs *5) causes VWD type 3 (VWD3).

**Fig. 1:**
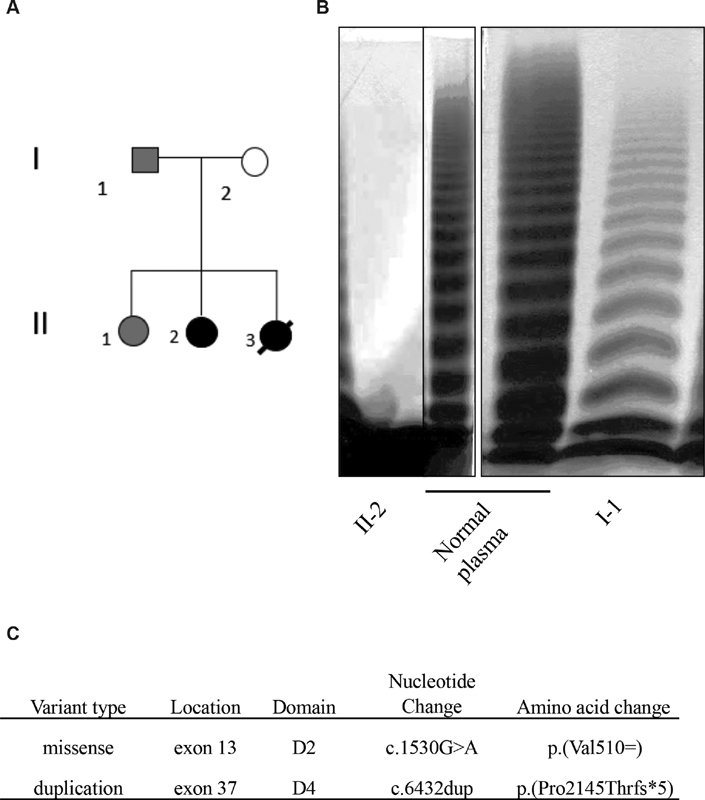
Presentation of a Martinican family with von Willebrand disease (VWD). ( **A** ) Proband II-2 genealogical tree. White symbol: VWD type 3 (VWD3) transmitter; gray symbol: patients VWD with p.(Val510 = ). ( **B** ) von Willebrand factor (VWF) multimer analysis in plasma from the proband (II-2) and her father (I-1). ( **C** ) Molecular analysis of the *VWF* gene of the proband by next-generation sequencing (NGS) IDT Sequencing.

An informed consent for a genetic analysis and phenotypic characteristics has been signed by all the patients included in this study. The *VWF* gene was analyzed by next-generation sequencing in 4 members of the original family that we have identified, that is, the father (I-1), the mother (I-2), the proband (II-2), and her sister (II-1) ( Fig. 1A ). The proband was a woman affected by severe hemorrhagic manifestations. Her biological profile was evocative of VWD3: VWF:Ag = 1 to 5%, VWF:RCo = 5%, FVIII:C = 2 to 3%, VWFpp = 6%, and a total absence of multimers assessed by electrophoresis ( Fig. 1B ). She was usually treated with plasma-derived VWF concentrates. Interestingly, the VWFpp level in this patient was higher than expected for typical VWD3. The sister (II-3) who died from a nonhemorrhagic cause had the same clinical-biological profile as the proband. *VWF* sequencing revealed the presence of two causative genetic variants present in a heterozygous state ( Fig. 1C ). The first one, p.(Val510 = ), is a previously unreported synonymous variant present in VWF propeptide, which is frequently found in Martinique, indicating the presence of a cluster. The second one, p.(Pro2145Thrfs*5), located on exon 37 has already been described in VWD type 1 (VWD1) patients 2 and leads to a shift of the reading frame and the appearance of a stop codon. In addition to these two variants, five variants/polymorphisms (p.Ala631Val, p.Met740Ile, p.His817Gln, p.Asp1472His, and p.Arg2185Gln) previously described as non- or little deleterious in healthy populations have also been detected in this patient.

We next studied the mother and the father of the proband. The mother (I-2) had a normal biological assessment (VWF:Ag = 131%, VWF:RCo = 116%) and was asymptomatic without any bleeding. Sequencing of the mother's *VWF* gene revealed a single variant on the VWF mature subunit: the c.6432dup which results in a stop codon. This molecular abnormality described in the mother (I-2) is in favor of a status of transmitter of VWD3. The father (I-1) experienced excessive bleeding only as a result of trauma or surgery. Sequencing of the father's *VWF* gene led to the identification of one potential causative variant: p.(Val510 = ), and five polymorphisms previously identified in healthy individuals. The phenotype reported by the father is of particular interest as biological assays did not report any dissociation between VWF:RCo (10%) and VWF:Ag (12%), suggestive of VWD1 whereas the study of plasma VWF multimers showed a significant and uniform reduction in the percentage of high molecular weight forms and intermediate molecular weight forms ( Fig. 1B ). This latter observation would be more compatible with a VWD type 2A (IIE) but the fact that the mutation is not in the D3 domain does not fit with such a picture. The second sister (II-1) of the proband has a clinico-biological phenotype similar to that of her father.

**Fig. 2:**
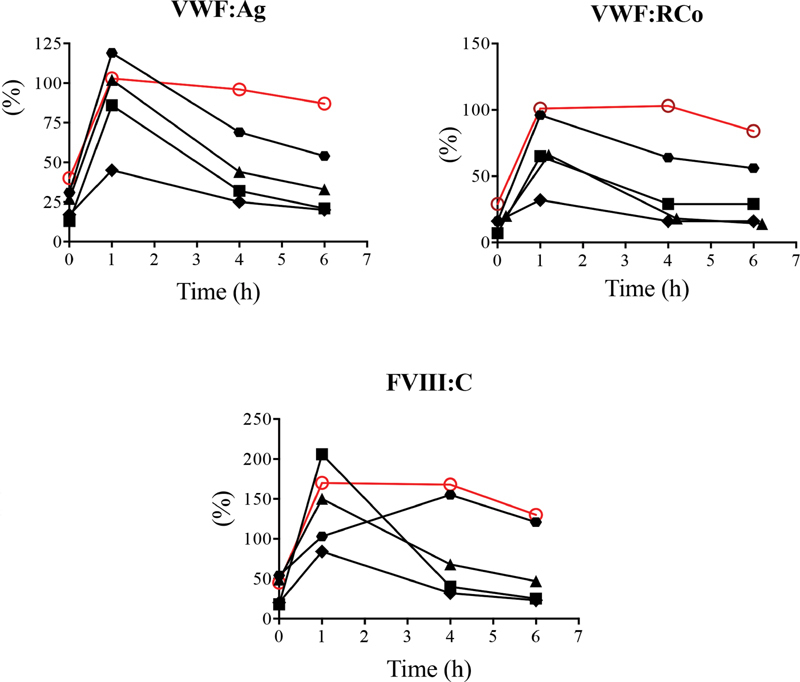
VWF:Ag, VWF:RCo, and FVIII:C levels after desmopressin (DDAVP) administration. Four patients carrying the p.(Val510 =) variant (black lines) and 1 von Willebrand disease type 1 patient (p.(Pro1413Leu)) were injected with DDAVP. VWF:Ag, VWF:RCo, and FVIII:C were measured at baseline and at 1, 4, and 6 hours post-DDAVP.

To understand better the effect of this new p.(Val510 = ) variant which appears to be relatively frequent in Martinique, we investigated 21 additional Martinican patients exhibiting the same variant. Patients' characteristics are indicated in Table 1 . Median age was 63 years (interquartile range [IQR], 45–77) and 65% were female. The striking feature in these patients was a significantly increased VWFpp/VWF:Ag ratio with a median of 5.62 (IQR, 4.36–6,14). Of note, this ratio could be calculated only for the 14 patients for whom the VWFpp level was measured. An increased VWFpp/VWF:Ag ratio (> 2.2) is indicative of an accelerated clearance of VWF. 3 To further investigate this potential mechanism, we decided to perform desmopressin (DDAVP) intravenous infusion (0.3 μg/kg) in 4 patients with the p.(Val510 = ) mutation and we measured VWF:Ag, VWF:RCo, and FVIII:C levels at different time points after infusion. The administration of DDAVP prompted a significant increase in VWF:Ag, VWF:RCo, and FVIII:C levels in these 4 patients as well as in a control, a VWD1 patient with the p.(Pro1413Leu) mutation, which does not lead to any clearance defect ( Fig. 2 ). Sixty minutes after DDAVP injection, the levels of VWF and FVIII:C decreased sharply, returning to baseline levels between 4 and 6 hours post-DDAVP for 3 patients out of 4 carrying the p.(Val510 = ) mutation. One patient (represented by the black hexagons on the figure) proved to be a better responder to DDAVP than the other 3 patients (despite the same molecular profile). However, even in this patient, VWF:Ag and VWF:RCo decreased quicker than for the control. These results strongly suggest that the p.(Val510 = ) variant induces an accelerated clearance of VWF.

**Table 1:** Clinical and laboratory characteristics of 23 Martinican patients with the p.(Val510 = ) variant

Characteristics	Patients p.(Val510 = ) ( *n* = 23)
Age, y	63 (45–77)
Females (%)	65
Blood group non-O (%)	30.43 a
FVIII:C (IU/dL)	26 (17–35)
VWF:Ag (IU/dL)	20 (16–24)
VWF:RCo (IU/dL)	14 (10–19)
FVIII:C/VWF:Ag ratio	1.4 (1–1.67)
VWF:RCo/VWF:Ag ratio	0.74 (0.5–0.94)
VWFpp/VWF:Ag ratio	5.62 (4.36–6.14) b

Note: Results are indicated as median (25th to 75th percentile) for age, FVIII:C, VWF:Ag, VWF:RCo, FVIII:C/VWF:Ag ratio, VWF:RCo/VWF:Ag ratio, and VWFpp/VWF:Ag ratio. Normal range for VWFpp/VWF:Ag 0.6–1.5.

a *n* = 7.

b *n* = 14.

**Fig. 3:**
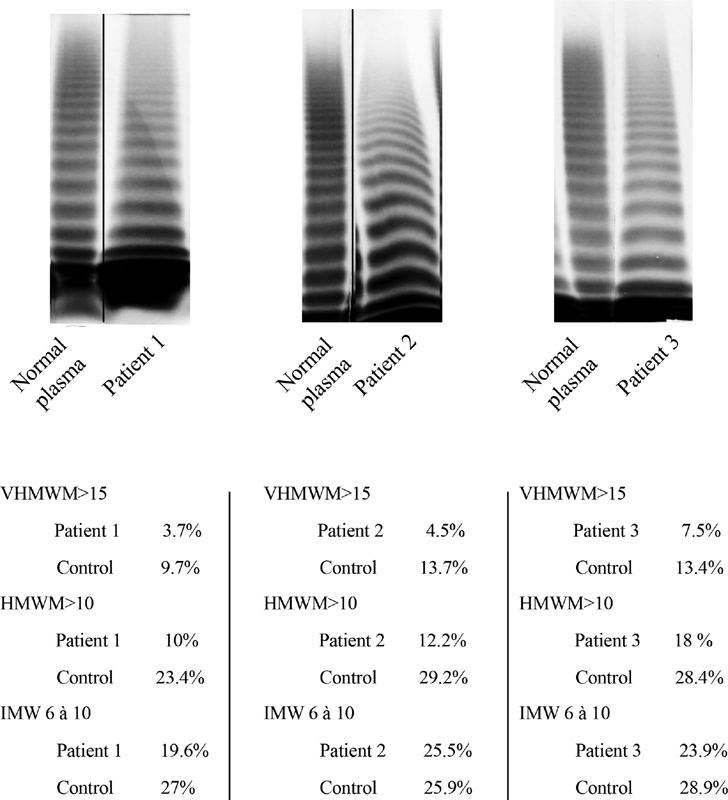
Plasma von Willebrand factor (VWF) multimeric analysis of 3 patients with p.(Val510 = ). Top panel: Each patient was analyzed on a separate gel and compared with normal human plasma run on the same gel. A black line indicates when the two samples were not run next to each other. Lower panel: Quantification of the multimers was done by densitometry. VHMWM, very high molecular weight multimers (>15 mers); HMWM, high molecular weight multimers (>10 mers); IMW, intermediate molecular weight multimers (6–10 mers).

Another interesting feature associated with this mutation is the difficulty to really assign the patients to a very specific VWD type or subtype. As already mentioned for the father of our original family, the multimeric profile indicates a variable loss of high molecular weight multimers ( Fig. 3 ) but biological measurements did not show any discrepancy between VWF:Ag and VWF:RCo in most cases. However, since the main effect associated with this mutation appears to be the clearance defect, we propose to classify the patients as belonging to the subtype 1C.

In conclusion, VWF mutational analysis can be valuable for diagnosing and investigating the molecular etiology of VWD. 4 The prediction softwares used (SpliceSiteFinder-like, MaxEntScan, GeneSplicer, NSPLICE, ESEFinder, RESCUE-ESE, and EX-SKIP) regarding c.1530G > A (p.(Val510 = )) interpreted the appearance of a donor site at 6 base pairs at the end of exon 13 which could potentially alter splicing. This synonymous mutation could therefore have an effect on VWF messenger ribonucleic acid processing, causing a shift in the reading frame and the appearance of a termination codon deletion of two codons. The American College of Medical Genetics and Genomics 5 predicted that this variant would probably be pathogenic. This study contributes to complete biological data on VWD, and more particularly on a population of Afro-Caribbean Martinican ancestry.
